# Correlation between measured oral health and oral health-related quality of life in people with epidermolysis bullosa: a prospective cohort study

**DOI:** 10.1186/s12903-024-05337-9

**Published:** 2024-12-19

**Authors:** Theresa Joanning, Guilherme Trento, Jeanette Köppe, Linda Daume, Johannes Kleinheinz, Marcel Hanisch, Ole Oelerich

**Affiliations:** 1 Department of Cranio-Maxillofacial Surgery, University Hospital Münster, Münster, Germany; 2 Institute of Biostatistics and Clinical Research, University of Münster, Münster, Germany; 3 Department of Prosthodontics, University Hospital Münster, Münster, Germany

**Keywords:** Epidermolysis bullosa, Oral health-related quality of life, Rare diseases, OHIP-14, PhOX

## Abstract

**Background:**

Inherited epidermolysis bullosa (EB) is a genetic disorder characterized by skin fragility and unique oral features. This prospective study aimed to analyze the correlation between oral health-related quality of life (OHRQoL) and the objectively measured oral health indices of people suffering from EB and within their subtypes.

**Methods:**

The German version of the Oral Health Impact Profile (OHIP-14G) was employed for the assessment of OHRQoL. Furthermore, all participants underwent a comprehensive clinical examination to evaluate their oral health via the Physical Oral Health Index (PhOX). Study participants were included through various self-help groups in Germany, regular appointments at the outpatient clinic for rare diseases with orofacial involvement, at the Department of Cranio-maxillofacial Surgery at the University Hospital Münster and through contact via social media.

**Results:**

A total of 33 individuals participated in the study. The study revealed that both OHRQoL and oral health were reduced. Individuals with dystrophic epidermolysis bullosa (DEB) were more negatively affected than were those in the junctional epidermolysis bullosa (JEB) and epidermolysis simplex (EBS) groups. The OHIP-G14 mean score of participants diagnosed with DEB was 25.2 (95% CI: 18.4–32.0; ± 13.7, range 5–49), and the PhOX mean score of DEB was 54.6 (± 15.7). A significant correlation was observed between PhOX and the OHIP score (rs = -0.54; *p* < 0.001).

**Conclusions:**

The findings of this study corroborate the challenges faced by patients with EB in the oral domain and the deterioration of their OHRQoL. These results emphasize the necessity for dentists to engage comprehensively in disease management, encompassing routine examinations, preventive dental care and oral hygiene education. Consequently, enhanced communication is required not only between dental and dermatological teams but also with caregivers.

**Supplementary Information:**

The online version contains supplementary material available at 10.1186/s12903-024-05337-9.

## Background

Epidermolysis bullosa (EB) is a rare, multiracial, inherited group of skin-related disorders that cause severe physical limitations in affected individuals [1, 2]. EB, classified as a mucocutaneous disorder, has no gender predilection and is estimated to affect approximately half a million people, the vast majority of whom are children [3, 4].

The particular fragility of the mucous and cutaneous membranes of these individuals, which characterize this condition, leads to the recurrent formation of vesicles and blisters in the presence of minor local trauma or even spontaneous damage. In certain cases, elevated room temperatures, routine tooth brushing and daily dental hygiene practices can potentially lead to the formation of vesicles [5, 6].

According to genetic studies, the pathogenesis of EB is related to several gene mutations and abnormalities in collagen, keratin and laminin proteins that alter cutaneous biology and affect cellular adhesion and integrity. There are four main types of EB based on the level of skin breakdown: EB simplex (EBS), junctional EB (JEB), dystrophic EB (DEB) and Kindler EB (KEB). Over thirty subtypes have been described [7, 8]. Each subtype has a different degree of phenotypic severity and a different impact on morbidity and mortality. Most people are affected by EBS (70%), DEB accounts for approximately 25%, JEB accounts for approximately 5%, and KEB is the rarest of the four major types of EB, with only 400 cases reported worldwide [9].

In the early 1990s, the National Epidermolysis Bullosa Registry (NEBR), after multicenter research and investigation, published guidelines for EB regarding its different subtypes and clinical, laboratory and diagnostic characteristics, as well as its oral manifestations [10]. Both mild and severe vesicles can affect the oral mucosa. The differences in and severity of the oral manifestations are strongly related to specific proteins that are abnormal or even absent [2]. Examples of such proteins include mutations in Keratin 5, Keratin 14, and Plectin, associated with EBS, as well as defects in Type XVII collagen and Laminin 332, linked to JEB, and the absence of Type VII collagen, which is characteristic of DEB [11]. Other oral findings include the absence of lingual papillae, ankyloglossia, erythema, milia, ulcers and tongue atrophy. The salivary glands are also affected, and it has been suggested that the absence of collagen VII during embryonic development leads to abnormal formation of glandular structures [12, 13].

Through this research, we wanted not only to explore the general oral health of people with EB but also to assess the impact of oral health on their quality of life (QoL). Little is known about the QoL perceptions of people with EB in relation to their oral health. Many studies have investigated only the QoL of people with EB in general [14–16]. The overall QoL of people with EB is negatively affected by different assessment tools, symptoms, physical aspects and psychosocial aspects [16]. In addition, there is a paucity of clinical studies on oral health characteristics and lesions in the literature, which include a significant number of patients [2, 12, 17].

No correlation has yet been found between objectively assessed and subjectively reported oral health, stated as H0: r = 0. Therefore, this study aims to validate and illustrate the association between oral health-related quality of life (OHRQoL) and oral health in people with EB.

## Materials and methods

### Study design and setting

The study was designed as a clinical examination preceded by the completion of questionnaires. Demographic information such as age, sex and current EB subtype, as well as the participant's general dental history, was recorded in free-text questions (see Supplementary File 1). The participants were given the OHIP-14G questionnaire, which is the German short form of the Oral Health Impact Profile (OHIP-49) questionnaire [18]. The validated PhOX questionnaire was used to assess patients’ oral health objectively [19].

### Ethical consideration

The study was approved by the Ethics Committee of Westfalen-Lippe (2021–683-f-S).

### Sample size determination

Assuming a two-sided significance level of 0.05 and a sample size of at least 40 participants, a Spearman correlation coefficient of r = 0.42 could be detected with a power of 81%. This finding was considered clinically relevant, as the correlation in healthy subjects was known from a previous study [19].

### Inclusion criteria

Participants were included in the study if they were at least 18 years old and had been diagnosed with EB. No other systemic or general illnesses resulted in the exclusion of the participant.

A consent form had to be signed beforehand. Only fully completed questionnaires were accepted (33 out of 33). Spelling and grammatical errors in free-text responses were not grounds for exclusion as long as the overall message could be conveyed.

### Data collection

Data were collected between 1 August 2022 and 1 July 2024. The datasets supporting the conclusions of this article are available upon request from the Department of Cranio-Maxillofacial Surgery, University Hospital Münster, Germany.

To reach as many people as possible for the study, various self-help groups in Germany were contacted. In particular, part of the data collection was carried out at DEBRA e.V. (Dystrophic Epidermolysis bullosa Research Association) self-help group meeting, in September 2022, in Rotenburg an der Fulda, Germany, as well as at a privately organized group meeting, in March 2023, in Willingen, Germany, and at the Alligatura (Alligatura Med. Consilium GmbH, Berlin, Germany) practical workshop, in February 2024, in Hannover, Germany. In addition, people were contacted via social media such as Instagram. Additional study participants were recruited through regular appointments at the outpatient clinic for rare diseases with orofacial involvement at the Department of Cranio-Maxillofacial Surgery, University Hospital Münster. Three dentists specializing in oral surgery and/or prosthodontics performed the clinical examination (T.J., M.H., O.O.). The calibration involved a joint, detailed review of each item on the questionnaire. The examiners performed the objective oral health assessment using the PhOX in each other’s presence to ensure the best possible harmonization of data collection between examiners.

### Physical Oral Health Index (PhOX)

The three calibrated investigators performed a clinical dental assessment of all study participants according to the PhOX [19], which uses a systematic approach to assess measurable oral and maxillofacial areas. Symptoms such as pain and/or paresthesia in the past 30 days were first recorded. Dental status was assessed via the decay‒missing‒filled teeth (DMFT) index adapted from the PhOX (see Reissmann et al. [19] for an explanation of the full index). To assess endodontic status, all teeth were examined for pain or discomfort on percussion. The pocket probing depth of the 6 Ramfjord teeth (16, 21, 24, 36, 41 and 44) was recorded to assess the periodontium [20]. Visual examination of the oral soft tissues included a comprehensive assessment for the presence of edema, erythema and loss of integrity. Saliva was assessed visually, quantitatively and qualitatively. The former included an assessment of oral mucosal moisture, while the latter differentiated between liquid and foamy secretions. Palpation was performed on the temporalis muscle, masseter muscle with 1 kg finger pressure, temporomandibular joint, parotid gland and submandibular gland with 0.5 kg finger pressure, according to the Diagnostic Criteria for Temporomandibular disorders (DC/TMD) protocol [21]. Pain intensity was rated on a scale of mild, moderate or severe, and pain familiarity was classified as known or unknown. Deviations from the ideal tooth, such as malocclusion, contact point deviation greater than 5 mm, nonideal tooth position, posterior crossbite, overjet and overbite, were identified. Normal oral function was assessed by measuring maximum active and passive mouth opening, even in the presence of pain. To ensure objectivity, each of the 14 subitems of the PhOX was assigned a score between zero and four. These subitems were weighted one, two or three times depending on their relevance, resulting in an overall score of 0 to 100, with 100 representing the objective best possible oral health (see Table 1).

**Table 1:** Physical Oral Health Index (PhOX) weights and ranges for each item

Item	Weight	Range
Number of teeth	3	0–12
Tooth Structure	3	0–12
Periodontium	3	0–12
Endodontia	2	0–8
Surface	1	0–4
Color	2	0–8
Moisturization	1	0–4
Pain on palpation	1	0–4
Continuity	1	0–4
Proportion	1	0–4
Mouth opening	1	0–4
Supporting Area	3	0–12
Pain	2	0–8
Paresthesia	1	0–4

### Oral Health Impact Profile (OHIP-14)

The German short form of the OHIP-14 was used to assess OHRQoL subjectively; it consists of 14 items related to the frequency of pain, limitations, social or physical distress, inconvenience and social life difficulties [18]. A score was assigned to each question on a Likert scale ranging from 0 (never), 1 (hardly ever), 2 (once in a while), 3 (often) to 4 (very often). Consequently, the overall OHIP score ranges from 0 to 56 points, with 56 representing the highest impact on OHRQoL. To ensure a consistent interpretation, it was essential to score the OHIP-14 according to the four dimensions of OHRQoL, as recommended [22]. These four dimensions—oral function, orofacial pain, orofacial appearance, and psychosocial impact—were each calculated from two of the OHIP-14 items. Consequently, the possible scores for each dimension range from 0–8, with 8 representing the worst possible outcome for that dimension of OHRQoL. The participants completed this questionnaire independently before the examination.

### Statistical analysis

The data were analyzed and presented via descriptive and graphical methods to convey both the oral health status and the perceived oral health quality of the subjects. The general demographic data (e.g., age, sex and age at diagnosis) of each participant and their subtypes (EBS, DEB, JEB) were analyzed descriptively. As the primary research question, the correlation between PhOX and OHIP was analyzed via the Spearman correlation coefficient. To illustrate the differences in the four dimensions of OHRQoL and the OHIP-14 score, the results are presented for each subtype and in total. In addition, differences between subtypes were tested via the Kruskal‒Wallis test. The same statistical method was used to present the differences in oral health. The PhOX results are also presented for each subtype and in total. For the primary research question, a p value < 0.05 was considered to indicate statistical significance (confirmatory). All other analyses were fully exploratory without adjustment for multiple testing and were interpreted accordingly. All the data were analyzed via SPSS Statistics for Windows version 29.0 (IBM Crp. Armonk, NY, USA) and RStudio version 2022.07.1 + 554 (RStudio PBC, Boston, MA, USA) for graphs.

## Results

### Age and time of diagnosis

A total of 33 people were included in this study. None had to be excluded. Among the 33 participants, 20 (61%) were female, and 13 (39%) were male. The mean age was 31.9 years (± 14.7), with the youngest participant being 18 years old and the oldest being 71 years old. Six participants were older than one year when they were diagnosed with EB. Detailed demographic information is shown in Table 2.

**Table 2:** Overview of the demographic data of each participant and their subtypes

	**Mean ***(SD)*	**Median ***(25. percentile – 75. percentile)*	**Range**	**EBS**	**JEB**	**DEB**
**Count** *n* (%)				7 (21%)	8 (24%)	18 (55%)
**Age** (years)	31.9 (± 14.7)	26 (20—44)	18 – 71	46.4 (± 17.9)	27.3 (± 11.1)	28.3 (± 11.4)
**Gender**						
Men				1 (14%)	3 (37%)	9 (50%)
Women				6 (86%)	5 (63%)	9 (50%)
**Age at diagnosis**	4.9 (± 12.8)	0.0 (0.0 – 0.0)	0—50	17 (± 22.3)	3.8 (± 10.6)	0.6 (± 1.9)

*EB * Epidermolysis bullosa in total, *EBS * EB simplex, *JEB * Junctional EB, *DEB * Dystrophic EB

### Subtypes of epidermolysis bullosa

Among the four known main groups of EB, three were found among our participants [23]. Among these patients, seven (21%) were diagnosed with EBS, eight (24%) with JEB and 18 (55%) with DEB. Patients with KEB were not represented in this study. The mean age at diagnosis for participants with EBS was 17 years (± 22.3), that for those with JEB was 3.8 years (± 10.6), and that for those with DEB was 0.6 years (± 1.9).

### OHRQoL

**Fig. 1:**
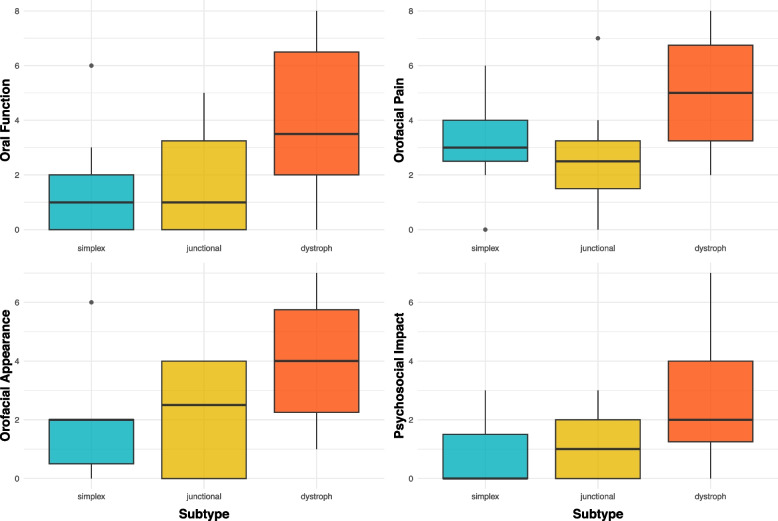
Boxplots for the four dimensions of oral health-related quality of life (OHRQoL) for each subtype

**Fig. 2:**
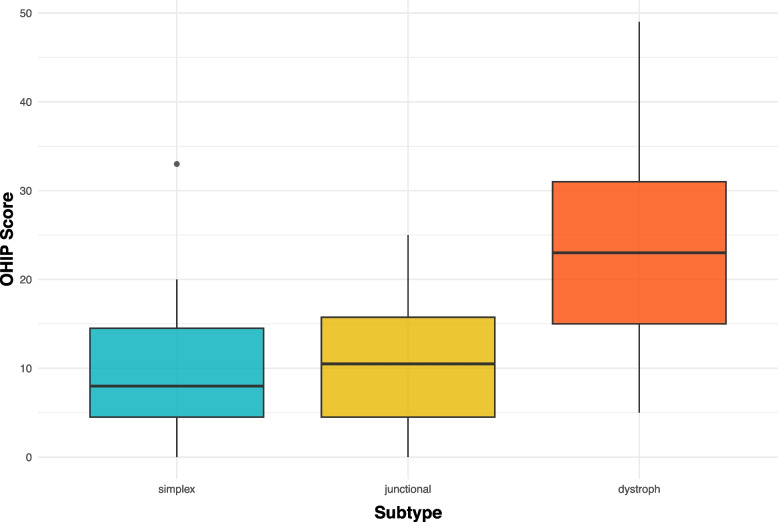
Boxplots for OHIP-G14 total scores for each subtype

The OHIP-14G score of all participants in this study was 18.7 (95% CI: 13.8–23.7; ± 13.9, range 0–49). Compared with the other two subtypes, the participants diagnosed with DEB had the highest OHIP-G14 mean score of 25.2 (95% CI: 18.4–32.0; ± 13.7, range 5–49) and the greatest impairment in the four dimensions ‘Oral function’ (4.0 ± 2.7), ‘Orofacial pain’ (5.1 ± 2.1), ‘Orofacial appearance’ (4.1 ± 2.0) and ‘Psychosocial impact’ (2.9 ± 2.0). Detailed information on the four dimensions for each subtype and for all the subjects is presented in Table 3 and Fig. 1. Compared with the other three domains, the dimension ‘Orofacial pain’, containing the answers to the two OHIP items ‘painful aching’ and ‘uncomfortableness to eat’, had the greatest impact on OHRQoL. (EB: 4.1 (± 2.3), EBS: 3.1 (± 2.0), JEB: 2.6 (± 2.3), DEB: 5.1 (± 2.1)). There were also noticeable differences in the OHIP-G14 total score among the different subtypes (*p* = 0.01) and for each domain between each predominant EB subtype (oral function (*p* = 0.04), orofacial pain (*p* = 0.03), orofacial appearance (*p* = 0.02) and psychosocial impact (*p* = 0.01)). Box plots for the OHIP-G14 total scores for each subtype are shown in Fig. 2.

**Table 3:** Scores (means ± SDs) for the four dimensions of the Oral Health-Related Quality of Life (OHRQoL) and Oral Health Impact Profile (OHIP-G14) summary score of each subtype were calculated

	**Oral function**	**Orofacial pain**	**Orofacial appearance**	**Psychosocial impact**	**OHIP-G14 Score**
**EB** (*n* = *33*)	2.9 (± 2.7)	4.1 (± 2.3)	3.1 (± 2.2)	2.1 (± 1.9)	18.7 (± 13.9)
**EBS** (*n* = *7*)	1.6 (± 2.23)	3.1 (± 2.0)	1.9 (± 2.0)	0.86 (± 1.21)	11.3 (± 11.5)
**JEB** (*n* = *8*)	1.8 (± 2.1)	2.6 (± 2.3)	2.1 (± 1.9)	1.1 (± 1.1)	10.6 (± 8.7)
**DEB** (*n* = *18*)	4 (± 2.7)	5.1 (± 2.1)	4.1 (± 2.0)	2.9 (± 2.0)	25.2 (± 13.7)
**p-Wert**	0.04	0.03	0.02	0.01	0.01

Differences between subtypes (*p*) were tested by Kruskal–Wallis test

*EB *Epidermolysis bullosa in total, *EBS* EB simplex, *JEB *Junctional EB, *DEB* Dystrophic EB

### Oral health

Among all the study participants, four had orthodontic treatment, and only three had removable dentures. None of these seven people had DEB. All participants reported oral involvement. A total of 18% had complete dentition (excluding third molars), and only one participant had neither filled nor missing teeth. All but one participant experienced changes in the surface of the oral mucosa, lip or tongue. Over 39% reported mouth pain at least several times a month. Fifteen out of eighteen patients with DEB and all with JEB had severely restricted mouth opening (< 30 mm), whereas all patients with EBS could open their mouth more than 30 mm. On the basis of the diagnostic findings, the PhOX score of all participants with EB was calculated, with a mean value of 62.0 (± 15.7, range 23–88; 95% CI: 56.5–67.6). The objectively measured oral health (PhOX score) of people with EBS had a higher overall score (75.4 (± 10.9)) than those with JEB (67.0 (± 9.1)) and DEB (54.6 (± 15.7)).

There were significant differences between the EB subtypes in four of the 14 PhOX score items (oral mucosal surface area (*p* = 0.01), jaw size ratio (*p* = 0.04), mouth opening capacity (*p* < 0.01) and pain frequency (*p* = 0.02)). Compared with those with the other subtypes, those with DEB had the lowest PhOX scores for these four items. Detailed information can be found in Table 4.

**Table 4:** Means and standard deviations for PhOX (Physical Oral Health Index) scores per subtype and total score

	**EB**	**EBS**	**JEB**	**DEB**	**p-Wert**
Teeth quantity	2.5 (± 1.0)	3.3 (± 0.5)	2.4 (± 0.9)	2.3 (± 1.1)	0.06
Condition of teeth	1.6 (± 1.1)	2.3 (± 1.0)	1.9 (± 1.4)	1.2 (± 0.9)	0.05
Condition of periodontium	1.7 (± 1.0)	1.3 (± 0.5)	1.9 (± 1.1)	1.8 (± 1.1)	0.34
Surface of oral mucosa	2.6 (± 1.2)	3.4 (± 0.5)	3.1 (± 0.6)	2.0 (± 1.2)	**0.01**
Condition of endodontium	3.8 (± 0.4)	3.7 (± 0.5)	3.9 (± 0.4)	3.8 (± 0.4)	0.75
Color and condition of oral mucosa	1.7 (± 1.5)	2.7 (± 1.6)	2.1 (± 1.6)	1.2 (± 1.3)	0.06
Moistening of oral mucosa	3 (± 1.2)	3.3 (± 1.1)	3.1 (± 1.1)	2.8 (± 1.2)	0.62
Pain on palpation of jaws and muscles	3.2 (± 1.3)	3.1 (± 1.5)	3.1 (± 1.3)	3.3 (± 1.3)	0.85
Continuity of jaws, palate and tongue	4.0 (± 0.0)	4.0 (± 0.0)	4.0 (± 0.0)	4.0 (± 0.0)	1.00
Size ratio of jaw	3.0 (± 1.3)	3.9 (± 0.4)	3.4(± 0.7)	2.6 (± 1.5)	**0.04**
Mouth opening capacity	1.3 (± 1.6)	2.7 (± 1.0)	1.8 (± 2.0)	0.5 (± 1.2)	** < 0.01**
Number of supporting zones	2.9 (± 1.4)	3.9 (± 0.4)	3 (± 0.9)	2.5 (± 1.7)	0.12
Pain frequency	2.5 (± 1.2)	3.3 (± 0.8)	3 (± 0.9)	1.9 (± 1.3)	**0.02**
Paresthesia frequency	2.8 (± 1.3)	3.4 (± 0.8)	3.1 (± 1.0)	2.4 (± 1.4)	0.22

Differences between subtypes (p) were tested by Kruskal–Wallis test

*EB* Epidermolysis bullosa total, *EBS* EB simplex, *JEB* Junctional EB, *DEB* Dystrophic EB

**Fig. 3:**
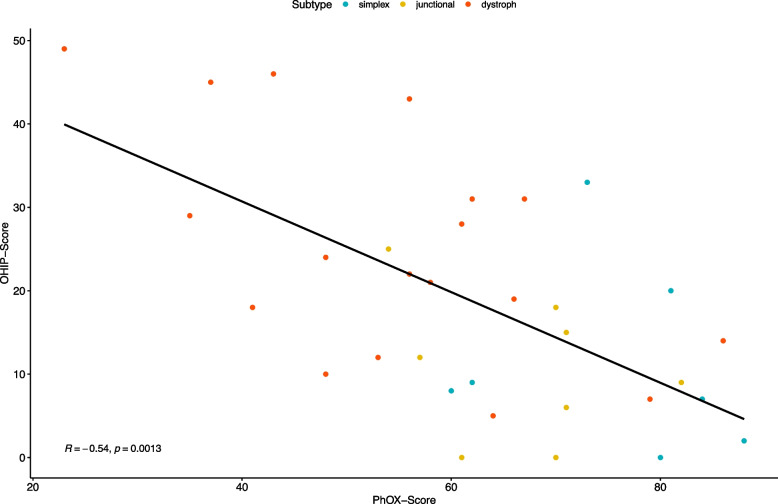
Scatterplot for correlation between PhOX (Physical Oral Health Index) and OHIP (Oral Health Impact Profile) scores with regression line; subtypes are coded by color

A significant correlation between PhOX and the OHIP score was observed (rs = -0.54; *p* < 0.001; see Fig. 2). A color-coded scatter plot for each subtype with a regression line is shown in Fig. 3.

## Discussion

This study is the first to investigate and compare both OHRQoL and objectively measured oral health in patients with EB. Our study established a correlation between these two variables within a single cohort. Although only 33 people with this rare disease participated, the results revealed a significant correlation between PhOX and OHIP scores; both OHRQoL and measured oral health were reduced. With a previous power analysis with assumptions based on the results of a prior study [24], we were able to obtain a significant correlation coefficient of r = 0.42 with a power of 81%. Despite the smaller number of cases (*n* = 33), a higher correlation coefficient (rs = -0.54, *p* = 0.0013) than originally expected was obtained.

The demographic information allows an understanding of the rarity of this condition and is the reason for the small sample size of this study. Rare diseases can have a detrimental effect on overall QoL, which may extend to OHRQoL [25]. A German study of people with rare diseases, which used the same questionnaire (OHIP-14) used in this study, reported mean scores ranging from 15.1–19.9, depending on the combination of symptoms. For diseases affecting the oral mucosa and periodontium, the score reached 19.7 [26]. This study revealed an average score of 18.7, with individuals diagnosed with DEB having a score of 25.2. The OHIP scores for each subtype are significantly higher than those in the general German population, where 90% of people (without dentures) have a score ≤ 11 [27].

Our results show that the OHRQoL of participants with DEB is significantly more impaired than that of participants with EBS and JEB. In terms of orofacial pain, function, appearance and psychosocial aspects, the DEB group was also significantly more affected than the other study groups were. Horn et al. [28] also investigated the difference in the QoL of general impairment between subtypes and concluded that participants with DEB were the worst affected.

While many authors, such as Horn et al., have assessed QoL in general and others have assessed different aspects of this rare disease, including oral and clinical manifestations and dental treatment options, this is the first study to assess individual OHRQoL in adults via the OHIP-14 and PhOX scores [16, 29]. Marty et al. conducted a similar study to investigate OHRQoL only but in children [29]. The OHIP questionnaire was adapted with figures and illustrations, and the age range of the participants was not the same. Nevertheless, the results are similar when comparing children with EB and healthy children. The author referred to the same three subtypes, with the majority being DEB (69%), as reported in this study (54.5%) [29]. Unfortunately, Marty et al. did not discuss the results between these three subtypes. Therefore, this study appears to be the only one that discusses QoL outcomes related to oral health among the three major subtypes in adults.

In addition, this study also revealed evidence of a general deterioration in oral health in patients with EB via PhOX examination. The PhOX score of all participants affected by EB was calculated, with a mean value of 62.0 (± 15.7), and DEB was calculated, with a value of only 54.6 (± 15.7). There is no comparable study in the literature that examines the oral health of people with EB via the PhOX questionnaire. However, it is a highly reliable and valid tool for assessing oral health [19]. Nevertheless, several studies have investigated the oral health of people with EB in general. These studies highlight significant oral complications, such as dental caries, enamel hypoplasia and other oral lesions, which vary depending on the type of EB [2].

Compared with those of EBS and JEB, the PhOX scores deteriorated in people with DEB. However, a closer look at the data revealed significant changes only in the oral mucosal surface area, mouth opening, pain frequency and jaw size ratio. For example, few differences were detected between the subtypes via qualitative and quantitative saliva analysis. In addition, all the participants seemed to have no major health problems with Salvia. Other authors also reported no difference in saliva analysis between the control and EB groups regarding salivary volume and salivary flow [2]. Furthermore, hyposalivation was not found in patients with EB, although changes in the glands could be demonstrated [2]. As in our study, the absence of collagen VII, which is thought to affect gland formation, did not affect salivation [30].

Yavuz et al. published clinical and oral findings in patients with EB in 2023 and reported that patients with EB have bullae and ulceration of the oral mucosa: patients with EBS are affected by only a few small bullae that heal without obvious scarring, and those with JEB are more likely to have oral lesions but also heal without scarring. Patients with DEB are extremely affected by extensive intraoral soft tissue involvement and lesion healing with scarring [31]. In our study, on average, more than half of the mucosal surface of patients with DEB was affected by superficial changes. Patients with EBS and JEB showed only small changes. However, in terms of the color and condition of the mucosa, our data did not show a similar significant difference between these subtypes, although participants with DEB were also more severely affected. It should be noted, however, that studies such as Yavuz’s do not report surface color as we did [31]. In general, scar-healing ulcers have a much greater risk of ankyloglossia, vestibule obliteration and microstomia [32]. In other studies, only patients with DEBs are affected by microstomia [31]. Our study showed different results. Patients with EBS and JEB were also affected by reduced mouth opening but less severely than patients with DEB. Twenty-three of all participants, or 70%, had a mouth opening of less than 30 mm. These results are consistent in that all groups also presented pathological changes in the mucosa, and these defects led to scarring and thus restricted mouth opening, as described above.

Looking more closely at the hard tissue, fewer than 20% of the participants in this study had a complete set of teeth. In addition, all of our participants had poor dental status. People with DEB had only slightly worse scores regarding the condition of teeth using the DMFT index, than did those in the other groups. Joseph et al. also reported the worst mean DMFT scores in patients with DEB (3.1 ± 5.3 vs. 3.9 ± 6.1) [33]. In addition, structural enamel defects, such as hypoplasia, have been documented in patients with EB and have been attributed to genetic mutations associated with the disease that also affect ameloblastic differentiation [13, 34]. According to Wright et al., JEB is the only subtype with structural enamel defects, but its composition may be normal to mildly altered [35]. Thus, our results did not reveal significantly worse tooth structure in the JEB group. However, caries is not a specific manifestation of EB but rather a consequence of the disruption of oral health habits [13, 35].

Nearly 40% of our participants experienced mouth pain at least several times a month. In general, oral conditions affect lifestyle; the absence of teeth results in difficulty chewing and an unattractive smile, which can contribute to social exclusion [14, 36]. It has been documented that people with EB express a strong need for tooth replacement for aesthetic and functional reasons [37], but limited mouth opening is a very challenging aspect of providing an adequate environment for dental treatment, including surgical and prosthetic procedures [38].

In our study, participants’ OHRQoL was most affected by orofacial pain. The calculated mean OHIP score was 4.1 (± 2.3) for all individuals with EB. Individuals with DEB had even worse results (5.1 (± 2.1)) than those with EBS and JEB did. Orofacial pain generally worsens dietary habits—too few cariogenic foods due to the necessity of soft diets and slower eating (because of marked oral blistering and ankyloglossia) —and worsens oral hygiene, which are predisposing factors for dental caries [32]. Caries leads to more pain and tooth loss if left untreated. Therefore, the optimal approach to help these individuals meet their needs is to improve their daily oral hygiene practices and increase professional preventive measures [39]. Rather, treatments and recommendations should be tailored to the individual subtypes.

Therefore, the goal of dental treatment in patients with EB is to preserve the dentition, avoiding invasive or even noninvasive treatments such as prosthetic rehabilitation. Oral treatment with conventional removable dentures is often associated with blistering due to friction of the denture on the mucosa. On the other hand, the installation of dental implants with a fixed prosthesis could increase patient comfort. However, surgical intervention is associated with anesthetic management. Local anesthesia may cause blistering, and intubation under general anesthesia may cause airway obstruction due to tissue damage and bullae formation. To prevent the formation of blisters, the anesthetic should be administered deeply within the tissue layers and injected slowly. This approach minimizes the risk of mechanical tissue separation caused by the solution [39]. In terms of general anesthesia Krämer et al. recommends to carefully consult patient’s physician and an anesthetic team with experience in EB [39]. In addition, the resorption of maxillary bone, which is common in patients with EB, requires bone reconstruction and augmentation, which requires delicate soft tissue manipulation [6, 17, 40–42]. The quality of life of these patients is clearly and severely compromised by these conditions.

Unfortunately, many dentists lack sufficient experience with rare diseases, which may limit their ability to provide appropriate care to these patients [43, 44]. A major focus should be on raising public awareness of rare diseases. Healthcare professionals should learn how to address rare diseases such as EB during their studies, not only through self-study or extensive literature review. This finding is in line with the findings of a study by Nguyen et al., which showed that OHRQoL improved when patients with a rare disease were treated by professionals with relevant expertise [45].

The main strength of this study is the groundbreaking discovery of the significant correlation between poor oral health and reduced QoL in patients with EB, with the greatest impact on the DEB group. The use of the PhOX examination supported the findings of the OHIP questionnaire and provided a better overview of the current dental status of these patients, particularly within the subtypes. This is an important step in improving dental treatment and thus the OHRQoL of people with EB. It is important to consider what appropriate dental care for EB patients should look like; therefore, larger, multicenter studies are needed to confirm these findings and explore the impact of oral health interventions on OHRQoL in EB patients. Studies should include a more diverse representation of EB subtypes, with a particular focus on underrepresented types such as KEB.

## Limitations

The main limitation of this study was the small sample size. The participants were only people with EB who were either members of a support group or had sought advice at the University Hospital of Münster. Future research with a larger cohort is needed to draw more robust conclusions. However, this is a challenge, particularly for studies focusing on rare diseases. A recent systematic review of twelve international studies on EB QoL reported an average sample size of 39.9 adults, with 33 participants in this study being reasonable. In Germany, approximately 2,400 people have EBS, and approximately 200 and 1000 have JEB and DEB, respectively [16, 46]. Another limitation is that not all four predominant subtypes could be represented in our study. People with KEB did not participate. However, this is also a challenge, as KEB is the rarest major subtype, with only 400 cases reported worldwide [9]. Although DEB is significantly less common in patients than EBS is, it can be assumed that patients with more severe impairment are more likely to seek help and meet in support groups [23]. However, this lack of diversity did not affect the results, as the aim was to gain insight into the oral health experiences of adults living with EB.

## Conclusions

This study revealed a significant correlation between subjectively measured OHRQoL and objectively measured oral health (PhOX), confirming our initial null hypothesis. In addition, there were marked differences in the results between each of the main groups (DEB, JEB and EBS). The participants with DEB had worse OHRQoL and physical oral health than did those with EBS and JEB. Patients with EB should be given more attention, oral hygiene should be improved, and dentists’ knowledge and training should be enhanced to improve oral health and thus the QoL of those affected. A clear distinction should be made between the subtypes. Larger case studies should be conducted in the future to gain a more accurate picture of the disease and the individual factors that negatively influence oral health.

## Supplementary Information

Supplementary Material 1.

## Data Availability

The datasets used and/or analysed during the current study are available from the corresponding author on reasonable request.

## Abbreviations

EB: Epidermolysis bullosa
EBS: Epidermolysis bullosa simplex
JEB: Junctional Epidermolysis bullosa
DEB: Dystrophic Epidermolysis bullosa
KEB: Kindler Epidermolysis bullosa
OHIP: Oral Health Impact Profile
PhOX: Physical Oral Health Index
QoL: Quality of Life
OHRQoL: Oral health-related quality of life
DC/TMD: Diagnostic Criteria for Temporomandibular disorders
