# Supplementary material for: Correlation between measured oral health and oral health-related quality of life in people with epidermolysis bullosa: a prospective cohort study
Source: BMC Oral Health. 2024 Dec 19;24:1501. doi: 10.1186/s12903-024-05337-9 (PMC11657462; doi:10.1186/s12903-024-05337-9)
Supplement: Supplementary file 1 — Supplementary Material 1. [file 12903_2024_5337_MOESM1_ESM.docx]

**Supplementary File 1: Translated Free-Text Questions**

1. How old are you?
2. What sex are you?
3. What subtype of epidermolysis bullosa do you have?
4. How old were you when you were diagnosed with the disease?
5. How much time has passed between the first appearance of symptoms and the definitive diagnosis of your disease?
6. Does your disease involve the oral cavity or the face? (e.g. missing teeth, anomalies in the shape of teeth, disproportion in the size and/or position of the upper and lower jaw, malformation of the dental hard tissue, cleft lip-jaw-palate, etc.) If yes, which?
7. Are you undergoing or have you undergone orthodontic treatment?
8. Do you wear dentures? (Bridge or denture?)
9. If so, is the denture removable?
